# Use of Fourier-Transform Infrared Spectroscopy With IR Biotyper® System for *Legionella pneumophila* Serogroups Identification

**DOI:** 10.3389/fmicb.2022.866426

**Published:** 2022-04-26

**Authors:** Maria Rosaria Pascale, Francesco Bisognin, Marta Mazzotta, Luna Girolamini, Federica Marino, Paola Dal Monte, Miriam Cordovana, Maria Scaturro, Maria Luisa Ricci, Sandra Cristino

**Affiliations:** ^1^Department of Biological, Geological, and Environmental Sciences, University of Bologna, Bologna, Italy; ^2^Microbiology Unit, Department of Experimental, Diagnostic and Specialty Medicine, University of Bologna, IRCCS S. Orsola-Malpighi University Hospital, Bologna, Italy; ^3^Bruker Daltonik GmbH, Bremen, Germany; ^4^Department of Infectious Diseases, Istituto Superiore di Sanità, Rome, Italy

**Keywords:** FTIR-spectroscopy, *Legionella pneumophila* serogroups, IR Biotyper®, diagnostics, environmental monitoring

## Abstract

*Legionella* spp. are Gram-negative bacteria that inhabit freshwater environments representing a serious risk for human health. *Legionella pneumophila* (*Lp*) is the species most frequently responsible for a severe pneumonia known as Legionnaires' disease. *Lp* consists of 15 serogroups (Sgs), usually identified by monoclonal or polyclonal antibodies. With regard to *Lp* serogrouping, it is well known that phenotyping methods do not have a sufficiently high discriminating power, while genotypic methods although very effective, are expensive and laborious. Recently, mass spectrometry and infrared spectroscopy have proved to be rapid and successful approaches for the microbial identification and typing. Different biomolecules (e.g., lipopolysaccharides) adsorb infrared radiation originating from a specific microbial fingerprint. The development of a classification system based on the intra-species identification features allows a rapid and reliable typing of strains for diagnostic and epidemiological purposes. The aim of the study was the evaluation of Fourier Transform Infrared Spectroscopy using the IR Biotyper® system (Bruker Daltonik, Germany) for the identification of *Lp* at the serogroup (Sg) level for diagnostic purposes as well as in outbreak events. A large dataset of *Lp* isolates (*n* = 133) and ATCC reference strains representing the 15 *Lp* serogroups were included. The discriminatory power of the instrument's classifier, was tested by Principal Component Analysis (PCA) and Linear Discriminant Analysis (LDA). All isolates were classified as follows: 12/133 (9.0%) as *Lp* Sg1 and 115/133 (86.5%) as *Lp* Sg 2–15 (including both ATCC and environmental *Lp* serogroup). Moreover, a mis-classification for 2/133 (1.5%) isolates of *Lp* Sg 2–15 that returned as *Lp* Sg1 was observed, and 4/133 (3.0%) isolates were not classified. An accuracy of 95.49% and an error rate of 4.51% were calculated. IR Biotyper® is able provide a quick and cost-effective reliable *Lp* classification with advantages compared with agglutination tests that show ambiguous and unspecific results. Further studies including a larger number of isolates could be useful to implement the classifier obtaining a robust and reliable tool for the routine *Lp* serogrouping. IR Biotyper® could be a powerful and easy-to-use tool to identify *Lp* Sgs, especially during cluster/outbreak investigations, to trace the source of the infection and promptly adopt preventive and control strategies.

## Introduction

*Legionella* genus includes bacteria that commonly inhabit fresh water environments where they are able to parasitize various protozoa species (Rowbotham, 1980; Fields, 1996; Fields et al., 2002). *Legionella* becomes a risk for human health when it spreads into man-made water systems, which act as amplifiers and disseminators of the microorganism representing the main sources of infection (Newton et al., 2010; Mercante and Winchell, 2015).

Although there has been only one report of infection transmitted from person to person (Borges et al., 2016; Correia et al., 2016), the inhalation of contaminated aerosol is the typical *Legionella* transmission route. Upon aerosol formation *via* man-made water systems, *Legionella* can infect the human lung and cause mainly two forms of disease: Pontiac fever, a non-pulmonary flu-like symptoms syndrome, and Legionnaires' disease, a severe pneumonia that can lead to death if specific antibiotic therapy is not promptly taken (Cunha et al., 2016).

*Legionella* virulence is due to its ability to infect and grow in human macrophages, while its environmental persistence largely depends on its ability to replicate inside protozoa and biofilms growth (Newton et al., 2010; Abu Khweek and Amer, 2018), and also to resist shock or continuous disinfection treatment applied to the water distribution systems. Host susceptibility is a crucial point for human infection: people with risk factors such as smoking, immune or chronic diseases, and age more than 50 years are more exposed to *Legionella* infections (Cunha et al., 2016).

Although 66 *Legionella* species have been documented (Parte et al., 2020), and about half of them are linked to human infections, *Legionella pneumophila* (*Lp*) is the most virulent and studied species. There are 15 distinct serogroups of *Lp* based on their reactions with antibodies that recognize the bacterial lipopylisaccharide (LPS) molecule (Ciesielski et al., 1986). In particular, according to epidemiological data, *Lp* serogroup 1 (*Lp* Sg1) is responsible for the majority of recognized Legionnaires' disease cases in Italy and in other European countries (European Centre for Disease Prevention Control, 2021; Rota et al., 2021a).

In Italy, the incidence of legionellosis in 2020 was 34.8 cases per million inhabitants, with a significant decrease compared to 2019, mostly due to the COVID-19 pandemic. Among the 2,074 surveillance notification forms sent to the National Register of legionellosis Surveillance relating to as many cases of legionellosis, 53 are reported as probable cases and 2,021 cases have been subsequently identified (Rota et al., 2021a).

Considering the European data, in 2019, 11,298 cases were reported, of which over 80% have been classified as confirmed and all ascribable to *Lp* Sg1 (European Centre for Disease Prevention Control, 2021). However, Legionnaire's disease remains widely underestimated because of the predominant usage of diagnostic methods that do not detect all *Legionella* species and serogroups.

According to the EU case definition, laboratory criteria for case confirmation must include at least one of the following three: (1) isolation of *Legionella* spp. from respiratory secretions or any normally sterile site; (2) detection of *Lp* antigen in urine; (3) significant rise in specific antibody level to *Lp* Sg1 in paired serum samples (European Commission, 2018).

The gold standard method to isolate *Legionella* both from clinical and environmental samples is the classical culture technique. Nevertheless, it is important to note that it is seldom performed in clinical laboratory practice not only due to the need of specific and expensive media but also to the unavailability of adequate specimens from the lower respiratory tract (Fields et al., 2002; Murdoch, 2003; Mercante and Winchell, 2015; Whiley and Taylor, 2016). Furthermore, after the isolation, serological tests such as the direct fluorescent antibody (DFA), indirect immunofluorescent assay (IFA), latex agglutination, immunochromatographic tests, etc, must be used. However, due to the frequent use of polyclonal antibodies, none of these tests provide highly specific and sensitive results for each of the *Lp* serogroups and *Legionella* non-*pneumophila* species (non-*Lp*).

Moreover, the 15 known *Lp* serogroups can be subdivided further into monoclonal subgroups. The specific monoclonal antibodies (MAbs) and /or *Legionella* serogroup rabbit antisera were introduced for serotyping. The most famous Dresden panel, based on monoclonal antibodies (MAbs) described by Helbig et al. (1997) and validated by European Study Group for *Legionella* Infections (ESGLI) (Helbig et al., 2002), represents a recognized approach for isolates serotyping. Unfortunately, the most precious monoclonal antibodies produced by the Dresden University are at a limited availability to be provided to all the laboratories (Helbig et al., 1997, 2002).

A novel method based on Real Time PCR to distinguish *Lp* and *Lp* Sg1in clinical specimens has been described in Mentasti et al. (2015), suggesting the possibility to introduce the detection of *Lp* nucleic acid among the criteria to define a LD confirmed case. This approach has been demonstrated to be very useful in outbreak events to discriminate Sg1 and the cluster of Sg 2–15 in recent Italian epidemic events (Scaturro et al., 2021). Unfortunately, these techniques are expensive and scarcely utilized by routine laboratories. Therefore, there is the need to find other strategies to discriminate *Lp* serogroups that overcome the limits of the current diagnostic tests.

The urinary antigen test (UAT) is a fast and non-invasive diagnostic method, but most of the commercial tests are highly specific only for *Lp* Sg1, and few of these tests show a cross-reactivity with the other serogroups (Helbig et al., 2001; Svarrer et al., 2012; Wong et al., 2021).

It is noteworthy that several severe cases of pneumonia due to other serogroups have been reported (Grottola et al., 2012; Nishizuka et al., 2014). Recently, an outbreak of community**-**acquired Legionnaires' disease caused by *Lp* Sg2 has been described in Italy (Scaturro et al., 2021). During this outbreak, the adoption of multiple diagnostic tests (culture, real-time PCR, and serology) made it possible to detect an otherwise unidentified epidemic event. Furthermore, the UAT poses a limitation into epidemiological investigation because it does not allow the correlation between clinical and environmental strains.

Molecular methods, such as PCR and real-time PCR, have improved the diagnosis enabling rapid and reliable identification of all *Legionella* species. Nevertheless, these techniques require a subsequent sequencing step to determine the serogroup, and they have a limited potential for routine application (Blyth et al., 2009). However, to date, a molecular standardized method for the discrimination of all different *Lp* serogroups has not been established (Mentasti et al., 2014).

In conclusion, for routine purpose, conventional methods are time-consuming and elaborate, often with a limited availability in routine laboratories (e.g., monoclonal antibodies, PCR or sequencing), therefore some handy and common tests (e.g., agglutination test) have reported low sensitivity and the occurrence of false negative results, leading to inaccurate *Legionella* identification (Fields et al., 2002; Pascale et al., 2020).

Recently, different rapid methods with high sensitivity and specificity have been developed to overcome the limitations of traditional methods for the detection and identification of several pathogens (Grenga et al., 2019).

Mass spectrometry is nowadays a widely used method to identify many microorganisms at species level, replacing traditional biochemical methods, and it is the reference method in clinical routine diagnostics (Rychert, 2019; Oviaño and Rodríguez-Sánchez, 2021). The MALDI Biotyper system (Bruker Daltonics GmbH & Co. KG) proved to be very effective for the identification of *Legionella* spp., although it requires the expansion of its library and fails to discriminate between *Lp* serogroups (Fujinami et al., 2011; Gaia et al., 2011; Svarrer and Uldum, 2012; Pascale et al., 2020).

Fourier Transform Infrared Spectroscopy (FTIR) has been proven to be an innovative and reliable approach for microbial typing. The technique enables the analysis of the biochemical composition of the microbial cells using the infrared light absorption, which is the result of the interaction of all the different (bio)molecules present in the cells with the infrared light (Martak et al., 2019). Several studies, performed with many different microbial genera and species, showed a high discriminatory power, enabling a differentiation at the species or subspecies level (Preisner et al., 2010).

The IR Biotyper® (Bruker Daltonics GmbH & Co. KG) is a FTIR-based system suitable for microbial typing in routine practice. Since its introduction, several studies investigated the use of IR Biotyper® to type different bacteria, especially enterobacteria and non-fermenting Gram-negative bacilli other than Gram-positive, such as *Streptococcus pneumoniae* and *Staphylococcus aureus* (Burckhardt et al., 2019; Martak et al., 2019; Rakovitsky et al., 2020).

On the contrary, to the best of our knowledge, only few and not recent studies have been performed for *Legionella* (in particular for *Lp*) identification using FTIR, and no studies have been carried out yet to develop a classifier based on an artificial neural networks (Horbach et al., 1988; Helm et al., 1991a,b).

In this study, different *Lp* serogroups, including American Typing Culture Collection (ATCC) reference strains and environmental isolates, were tested by using the IR Biotyper® classifier discriminatory power to establish the ability of this system to differentiate the single *Lp* serogroups. The results were compared with the traditional latex agglutination test, and the limitations and potential of this methodology to lay the foundation for *Legionella* classifier improvement of an artificial neuronal network development were discussed.

## Materials and Methods

### Isolates Collection of *Lp* Serogroups

A total of 133 *Lp* isolates representing all the 15 different serogroups were included in the study.

In detail, 15 isolates from *Lp* Sg1 to *Lp* Sg15 were American Type Culture Collection (ATCC) reference strains. The remaining isolates (n = 118) were all environmental strains selected from a *Legionella* collection, stored in our laboratory, and originated from routine sampling, in accordance with the *Legionella* surveillance program established in Italy (Italian National Institute of Health, 2015).

### *Legionella pneumophila* Culture and Serogrouping by Latex Agglutination Test

The *Lp* isolates come from hot- and cold-water environmental samples collected in hospital, health-care facilities, companies, homes, hotel, and spa facilities from 2014 to 2021.

The *Legionella* culture was performed according to ISO 11731:2017 (International Organization for Standardization, 2017), and the isolates were identified by latex agglutination test (*Legionella* latex test kit, Thermo Fisher Diagnostic, Basingstoke, UK), a test able to discriminate between *Lp*1, *Lp* Sg 2–14, and *Legionella* non-*pneumophila* species. In addition, the polyclonal latex reagents test was used to determine the precise serogroup of the *Lp* Sg 2-14 strains (Biolife, Milan, Italy). The latex agglutination test is based on an antigen–antibody reaction using latex particles sensitized with antibodies that, in presence of specific *Legionella* cell-wall antigens, agglutinate to form visible clumps, which is considered a positive result.

All strains were unfrozen and sub-cultivated on buffered charcoal yeast extract (BCYE) agar plates with (cys +) and without (cys -) L-cysteine (L-cys) supplementation (Thermo Fisher Diagnostics, Basingstoke, UK) and incubated for 48 ± 2 h with 2.5% CO_2_ to test their viability. Moreover, as negative control, the same isolates were streaked onto tryptone soy agar (TSA) with 5% sheep blood agar (Thermo Fisher Diagnostics, Basingstoke, UK) and incubated under the same conditions previously described, considering that *Legionella* does not to grow on TSA with 5% blood agar. Only the colonies that grew on BCYE cys + agar were then tested by IR Biotyper®.

The relative abundance of each serogroup involved in the analysis reflects their local prevalence based on environmental surveillance program (unpublished data).

Table 1 shows the isolate numbers of *Lp* environmental and ATCC serogroups involved in the study that were characterized by the agglutination test.

**Table 1:** The number of environmental and ATCC *Lp* serogroups isolates included in the study.

	***Lp* serogroups (Latex agglutination test)**	**Number of isolates (n)**
	Sg1—ATCC 33152	1
	Sg2—ATCC 33154	1
	Sg3—ATCC 33155	1
	Sg4—ATCC 33156	1
	Sg5—ATCC 33216	1
	Sg6—ATCC 33215	1
ATCC strain	Sg7—ATCC 33823	1
*Lp* serogroups	Sg8—ATCC 35096	1
(*n* = 15)	Sg9—ATCC 35289	1
	Sg10—ATCC 43283	1
	Sg11—ATCC 43130	1
	Sg12—ATCC 43290	1
	Sg13—ATCC 43736	1
	Sg14—ATCC 43703	1
	Sg15—ATCC 35251	1
	Sg1	11
	Sg2	9
	Sg3	13
	Sg4	5
	Sg5	7
	Sg6	13
Environmental strain	Sg7	2
*Lp* serogroups	Sg8	13
(*n* = 118)	Sg9	10
	Sg10	8
	Sg11	6
	Sg12	6
	Sg13	8
	Sg14	7

### Sample Preparation for IR Biotyper® Analysis

The growth of fresh isolates on BCYE cys + for 48 hat 35 ± 2°C and with 2.5% CO_2_ have been directly processed on IR Biotyper®. Sample preparation was performed following the manufacturer's instruction. For the FTIR spectroscopy analysis, the environmental isolates were analyzed as one biological replicate, while the ATCC strains, due to the presence of only one isolate for each *Lp* serogroups, were analyzed as three biological replicates. Briefly, an amount of two overloaded 1-μl loops of bacterial colonies were resuspended into 50 μl of 70% ethanol solution in an IR Biotyper® suspension vial, which contains metal cylinders for an optimal homogenization of the sample. After mixing, 50 μl of sterile water was added. Bacterial suspension (15 μl) was spotted in four technical replicates on the 96-spots silicon IR Biotyper® sample plate and were left dry for 15–20 min at 35 ± 2°C. As quality control, two IR Test standards (IRTS1 and IRTS2), spotted in duplicate, were also included.

### Evaluation of the IR Biotyper® *Legionella* Automated Classifier

The *Lp* classifier included in the IR Biotyper® software enables the automatic (i.e., immediately after spectra acquisition) classification of *Lp* in two classes, Sg1 or Sg 2–15. The classifier consists of an artificial neural network (ANN) trained with well-characterized isolates, as described by the manufacturer. The ANN predicts the class of unknown samples based on learnt discriminatory features. Additionally, the classifier evaluates the similarity of the unknown sample spectra with the training spectra. It calculates the outlier factor (i.e., the spectral distance of the tested isolates with the spectra of the isolates used to train the classifier) that enables to provide a traffic light color code result by applying threshold values defined by the distribution of outlier values of the validation cohort of samples. A classification result with a green (outlier value <2.00) code means that the spectra of the tested sample are in the same spectral space of the strains used to train the classifier. A classification result with a yellow code (outlier value 2.00–2.99), means that the spectra of the tested sample are at the periphery of the spectral space of the strains used to train the classifier. A classification result with a red code (outlier value > 3.00) means that the spectra of the tested sample falls out of the spectral space of the strains used in the training set, therefore the result can be not-reliable. The cut-off values used for thresholds (2.00 and 3.00) were established by analyzing the distribution of the outlier values of the external samples' dataset (which included isolates which were not part of the training set, including non-*Lp*) and choosing values considering the Youden index. The red threshold selected proved to safely exclude all non-*Lp* isolates.

### Spectra Acquisition and Analysis

Spectra were acquired and processed by the OPUS software V7.5.18 (Bruker Optics, GmbH) and the IR Biotyper® Client Software V3.0 (Bruker Daltonik GmbH), with the default settings recommended by the manufacturer. The acquisition was in the wave number range between 4,000 and 500 cm^−1^. The second derivative was calculated, and data corresponding to the polysaccharide's absorption region (1,300–800 cm^−1^) were vector normalized. Similarity analysis was determined by principal component analysis (PCA) and linear discriminant analysis (LDA).

### Hierarchical Cluster Analysis

For Sg1, Sg7, and Sg11, an exploration of the presumptive relatedness among single isolates was explored by HCA, comparing IR Biotyper® clustering with the source of the isolates and the year of isolation. HCA was performed by the IR Biotyper® software version 3.0 (Bruker Daltonik, Germany) using Pearson's correlation to calculate the similarity matrix and average linkage (also known as unweighted pair group method with arithmetic mean, UPGMA) for agglomerative clustering. The nested clusters were displayed in the form of a dendrogram. A plausible cut-off value for partitioning was automatically calculated by the software using a supervised approach. This was done with the “SDI × mC” algorithm, which maximizes the product of Simpson's index of diversity (SDI, ranges from 0 to 1), calculated from partitioning the whole dendrogram, and the mean coherence (mC) of all predefined groups [by default, the groups are defined by the isolates, but also other grouping could be done (e.g., serogroups)]. The clustering cut-off differs among the different species of microorganisms, on the basis of the biological variance of each species, on the level of discrimination investigated, and in relation to the statistical method used to perform cluster analysis (e.g., metric, linkage type).

To perform the LDA as spectra pre-processing, the minimum number of isolates necessary was achieved by adding an isolate as the external negative control.

## Results

### IR Biotyper® Automated Classifier Results

Currently, IR Biotyper® automated classifier is able to differentiate *Lp* Sg1 from *Lp* Sg 2–15, providing a result in terms of class (Sg1 or Sg 2–15), and reliability score (a color-code) is based on the spectral distance of the acquired spectrum to the spectra used to train the classifier. Therefore, the results returned by software were compared with the identification already obtained by serological agglutination test.

The IR Biotyper® automated classifier was able to correctly classify 12/133 (9.0 %) *Lp* Sg1 isolates and 115/133 (86.5%) isolates as *Lp* Sg 2–15 (including both ATCC and environmental *Lp* serogroup isolates). Moreover, the mis-classification of 2/133 (1.5%) of *Lp* Sg 2–15 isolates, which then returned as *Lp* Sg1 was observed, and 4/133 (3.0%) isolates were not classified.

Regarding the traffic light color code results, 112/133 isolates (84.2%) were classified with green score, 17/133 (12.8%) with yellow score, and 4/133 (3.0%) delivered a red score result. Among the yellow score, 15/17 were actually correctly classified, while 2/17 were confirmed as wrongly classified, considering the previous identification obtained with agglutination test.

More in detail, among the environmental isolates (*n* = 118) all the *Lp* Sg1 (11/11) were correctly classified by the instrument with a green score.

The remaining *Lp* Sg 2–15 isolates (*n* = 107) were classified with a green score as follow: 9/9 as *Lp* Sg2 and 13/13 as *Lp* Sg3. Among the five isolates of Sg4, 3/5 were correctly classified with a green score and 2/5 with a yellow score. The seven isolates of Sg5 were classified as follows: 6/7 with green score and 1/7 with yellow score. All the 13 Sg6 isolates were correctly classified with green score. Regarding the classification of the two isolates of Sg7, despite the assignment of a yellow score, one was correctly classified as Sg7, while the other one, was mis-classified as Sg1.

The isolates belonging to Sg8 were all correctly classified with a green score. Nine of the ten isolates belonging to Sg9 were classified with a green score, while 1/10 as a yellow score. The Sg10 isolates were classified as follows: 7/8 with a green score and 1/8 with a yellow score. One of the six isolates of Sg11 was classified with a red score, 3/6 with a green score, and 2/6 with a yellow score. Most of the Sg12 isolates (5/6) were classified with a green score and only one (1/6) with a yellow score. Regarding the classification of Sg13, we obtained 6/8 isolates with a green score, 1/8 with a yellow, and 1/8 with a red score. Finally, 5/7 Sg14 isolates were classified with a green score, 1/7 was classified with a yellow score, and 1/7 with a red score.

The IR Biotyper® classifier correctly recognized the three biological replicates of each ATCC *Lp* serogroup with a green and yellow score, while only the *Lp* Sg10 ATCC isolate was classified with a red score. Again, a mis-classification was observed for the ATCC strain belonging to Sg 7 that was mixed up in Sg1.

All the discrepancies results, were retested by agglutination test, confirming the identification performed during the isolates selection.

As a consequence, the automated classifier showed an accuracy of 95.49% (127/133) and an error rate of 4.51% (6/133). Sensitivity and specifity for *Lp* Sg1 were: 100 and 98.35%, while for *Lp* Sg 2–15 they were 95.0 and 100%, respectively.

Table 2 shows the schematic representation of serogrouping obtained for each isolate belonging to environmental and reference strains.

**Table 2:** *Lp* serogroups classification results.

	***Lp* serogroups (Latex agglutination test)**	**Total of isolates (*n*)**	**IR Biotyper® classification results** **(*****Lp*** **Sg1 vs**. ***Lp*** **2–15)**			
			**Green (0.00–1.99)**	**Yellow (2.00–2.99)**	**Red (>3)**	**Mis-classification with other serogroups**
	Sg1—ATCC 33152	1	1			
	Sg2—ATCC 33154	1		1		
	Sg3—ATCC 33155	1	1			
	Sg4—ATCC 33156	1	1			
	Sg5—ATCC 33216	1	1			
	Sg6—ATCC 33215	1	1			
ATCC strain	Sg7—ATCC 33823	1		1		Returned as Sg1
*Lp* serogroups	Sg8—ATCC 35096	1		1		
	Sg9—ATCC 35289	1		1		
	Sg10—ATCC 43283	1			1	
	Sg11—ATCC 43130	1		1		
	Sg12—ATCC 43290	1	1			
	Sg13—ATCC 43736	1	1			
	Sg14—ATCC 43703	1	1			
	Sg15—ATCC 35251	1	1			
	Sg1	11	11			
	Sg2	9	9			
	Sg3	13	13			
	Sg4	5	3	2		
	Sg5	7	6	1		
	Sg6	13	13			
Environmental strain	Sg7	2		2		1 returned as Sg1
*Lp* serogroups	Sg8	13	13			
	Sg9	10	9	1		
	Sg10	8	7	1		
	Sg11	6	3	2	1	
	Sg12	6	5	1		
	Sg13	8	6	1	1	
	Sg14	7	5	1	1	

*The green, yellow, and red colors represent the classification score returned by IR Biotyper® classifier*.

### IR Biotyper® Similarity Analysis

**Figure 1:**
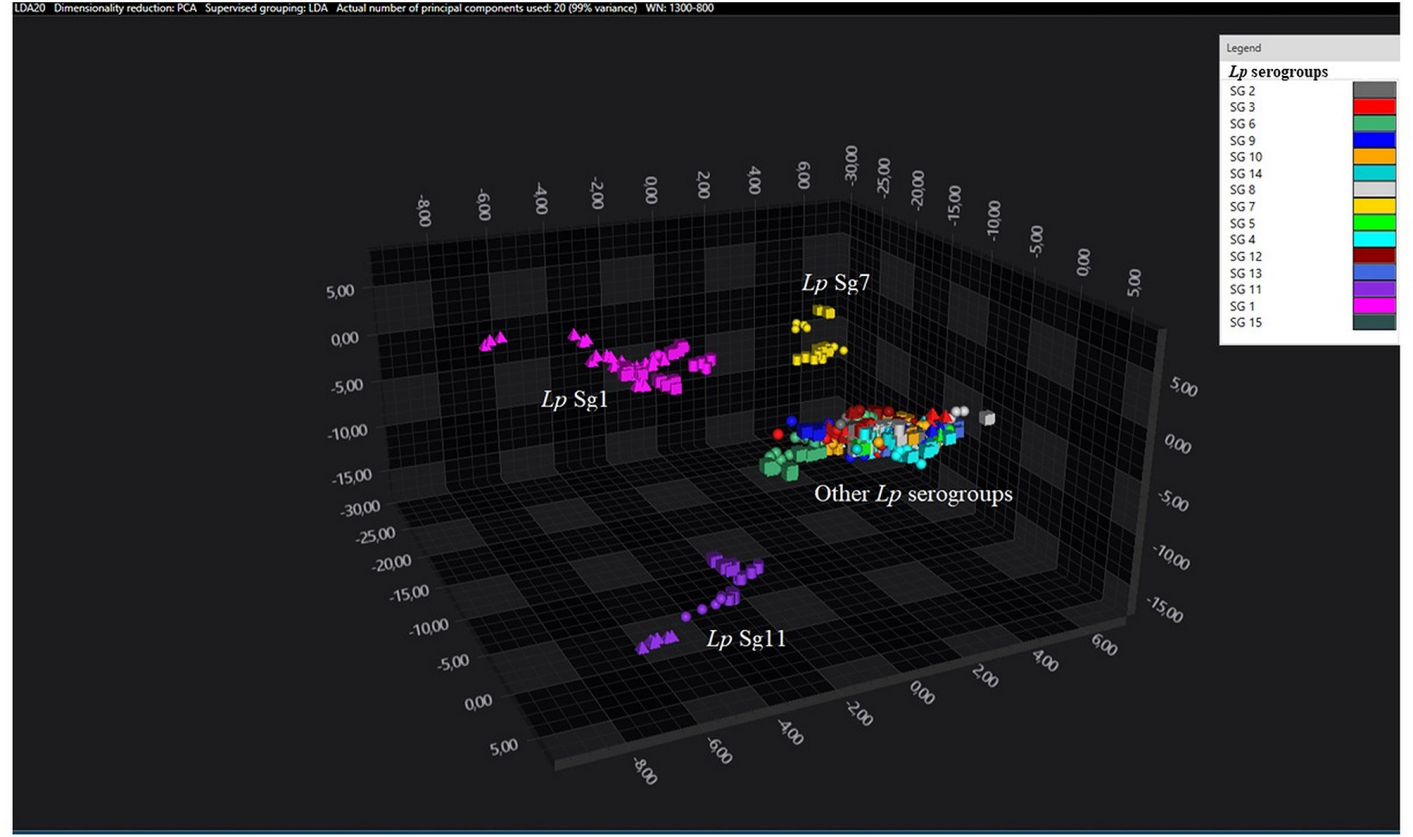
3D scatter plot representation of ATCC and environmental isolates. LDA was performed with 20 PCs. The single circle, triangle, or square represent one single spectrum (biological and technical replicates). Each serogroup is indicated by a different color. The separation of Sg1 (pink), Sg7 (yellow), and Sg11 (purple) from the other serogroups is well visible.

**Figure 2:**
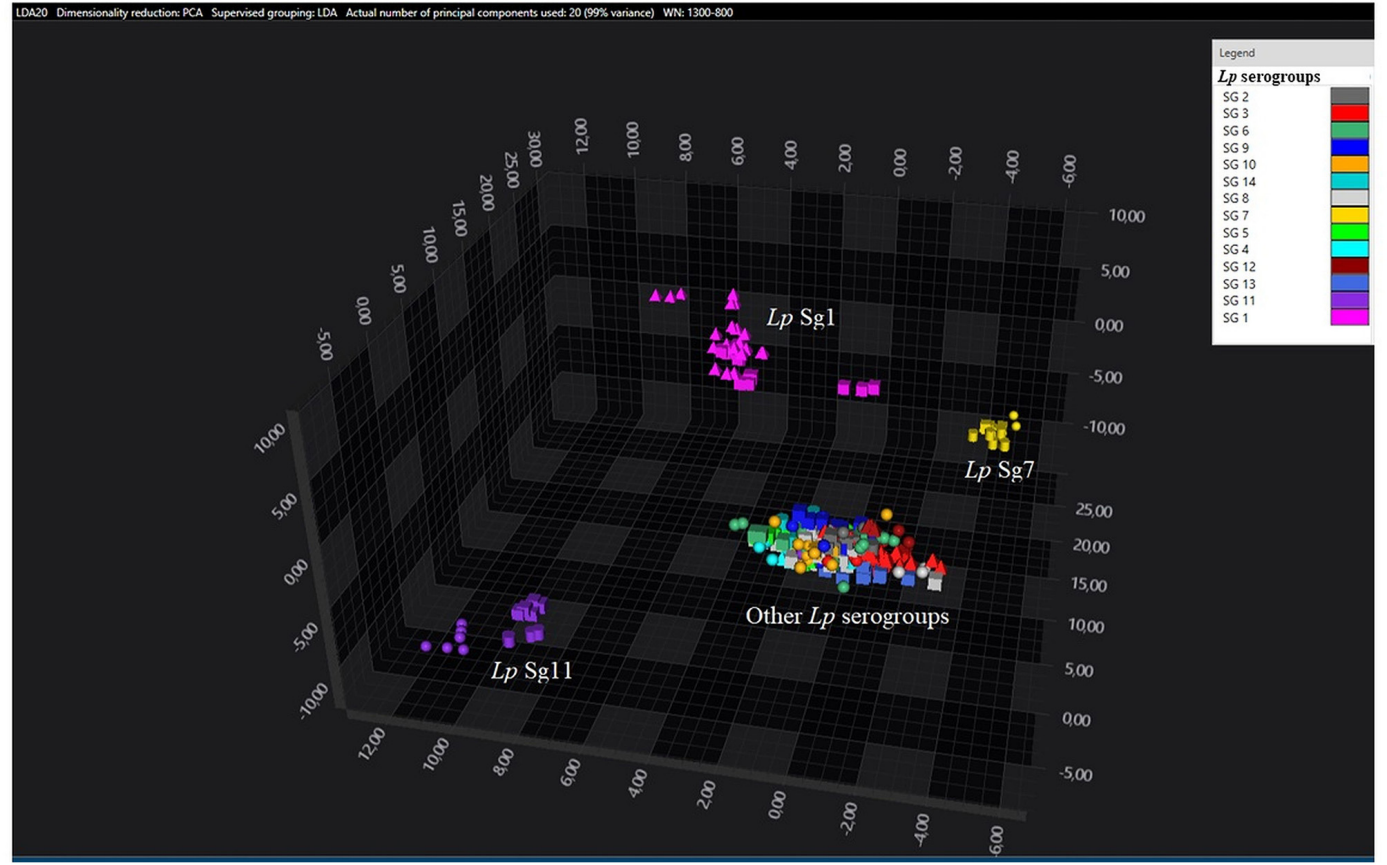
3D scatter plot of *Lp* environmental isolates. LDA was performed with 20 PCs. The single circle, triangle, or square represent one single spectrum for each isolate (technical replicates). Each serogroup is indicated by a different color. The separation of Sg1 (pink), Sg7 (yellow), and Sg11 (purple) from the other serogroups is well visible.

**Figure 3:**
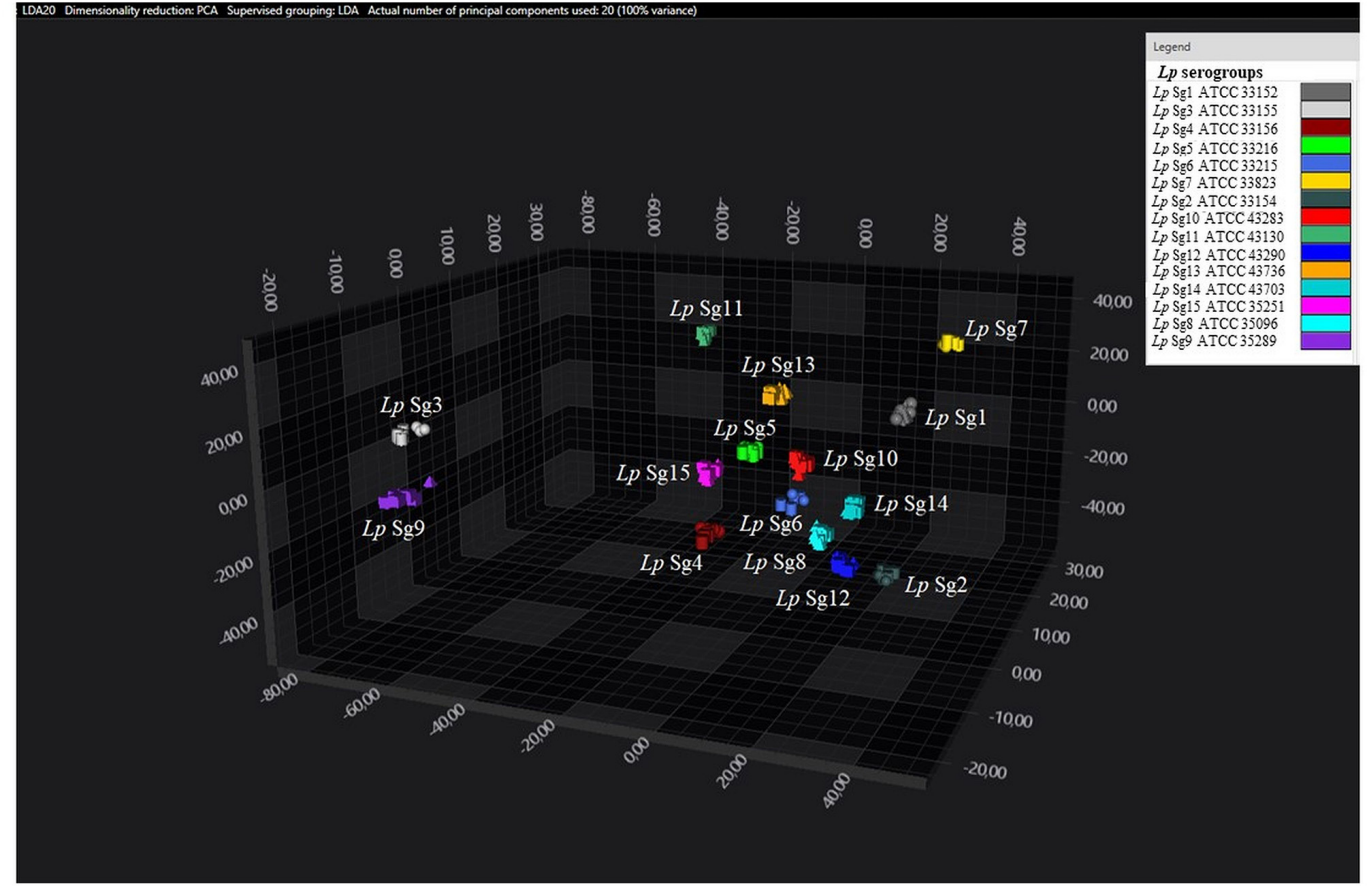
3D scatter plot of *Lp* ATCC reference strains. LDA was performed with 20 PCs. Each single circle, triangle, or square represent one single spectrum for each isolate (biological and technical replicate). Each serogroup is indicated by a different color.

**Figure 4:**
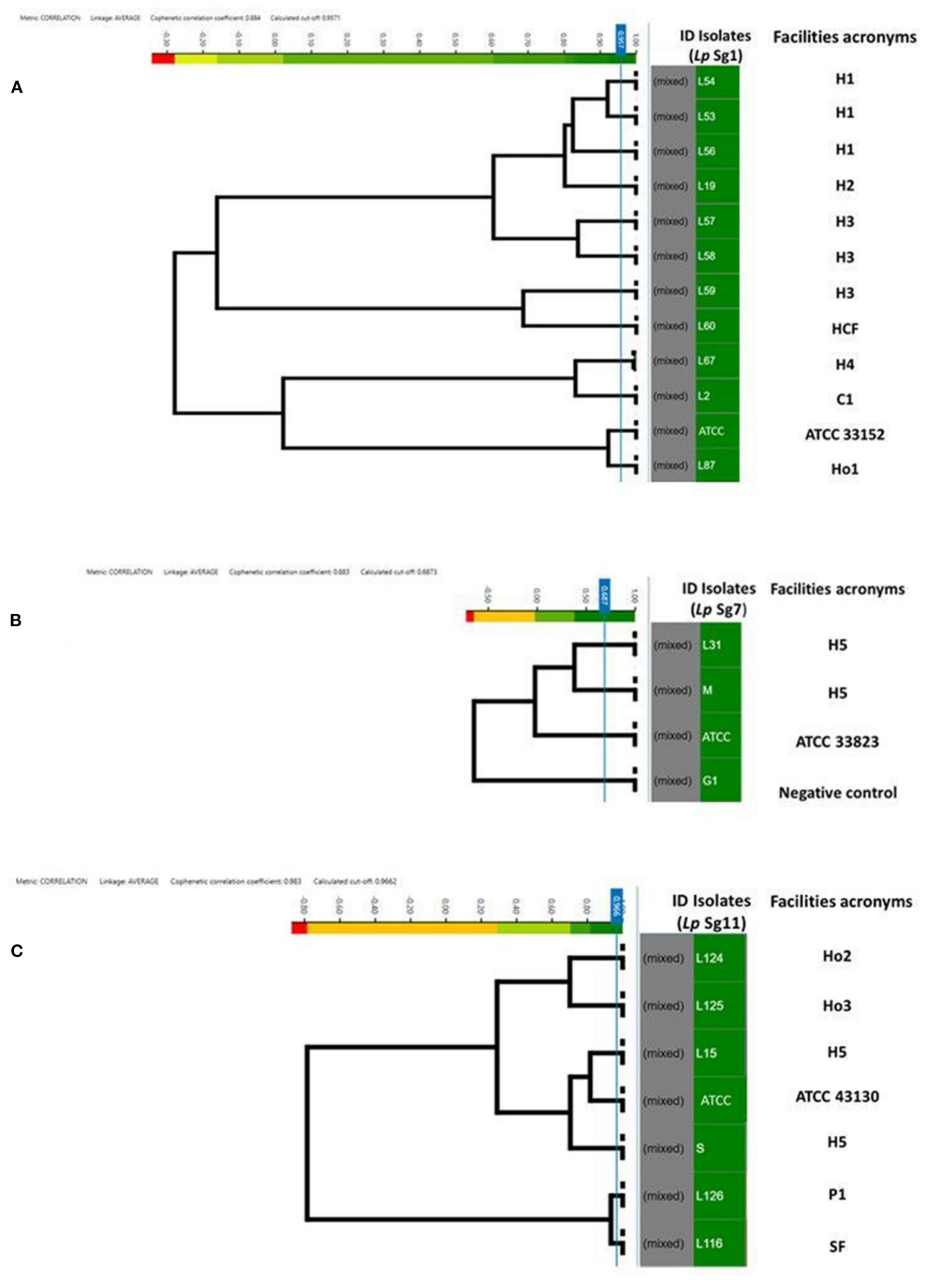
Clustering of IR Biotyper® by HCA, developed for dendrograms: *Lp* Sg1 **(A)**, *Lp* Sg7 **(B)**, *Lp* Sg11 **(C)** isolates. In the gray column the term “mixed” is referred to the merging of spectra obtained for each isolate. In the green column the isolates ID. The NEGATIVE control, was represented by *Lp* Sg4 isolates **(B)**. The cut-off value was automatically calculated by instrument software. The “Facility acronyms” used as follows: H, hospital; HCF, health care facilities; C, company; SF, Spa facility; Ho, hotel; P, private homes.

Principal component analysisand LDA were performed using 20 principal components (PCs) to achieve 99% of variance. The results of PCA and LDA for the different *Lp* serogroups are shown in the Figures 1–4. The two approaches were chosen to visualize the clustering.

Consistent with the results of the classifier, the following figures show four different well separated clusters where it is possible to distinguish Sg1 (pink), the Sg7 (yellow), and the Sg11 (purple), as opposed to the other Sgs that appear indistinguishable.

Noteworthy, the *Lp* ATCC strains are all well separated from each other. Specifically, Figure 1 represents the 3D scatter plot result obtained by PCs analysis for all the isolates involved in this study. Figures 2, 3 show the PCS 3D scatter plot results obtained by distinguishing the environmental isolates from *Lp* ATCC references strains.

### Analysis of *Lp* Sg1, Sg7, and Sg11 Clustering Spectra Using Different Cutoff Values

To assess the similarity and software ability to recognize and clusterize each other, a dendrogram of the *Lp* Sg1, Sg7, and Sg11, clearly separated from each other and from other *Lp* serogroups, was built (Figures 4A–C). The isolates came from different facilities labeled as follows: hospitals (H), health care facilities (HCF), hotels (Ho), spa facilities (SF), and companies (C) moreover they were collected in different years. All of them were then compared with the respective *Lp* ATCC serogroup isolate.

The ID of the isolates, source and year of isolation, and the acronym used are listed in Table 3.

**Table 3:** Isolates ID with their source, year of isolation, and respective acronyms reported in the dendrograms.

***Lp serogroups***	**ID on the dendrogram**	**Year and source of the isolation**	**Facilities acronyms**
Sg1	L53	2014—Hospital	H1
	L54	2015—Hospital	
	L56	2016—Hospital	
	L19	2015—Hospital	H2
	L57	2014—Hospital	H3
	L58	2015—Hospital	
	L59	2015—Hospital	
	L60	2015—Health Care Facility	HCF
	L67	2014—Hospital	H4
	L2	2020—Company	C1
	L87	2021—Hotel	Ho1
Sg7	L31	2020—Hospital	H5
	M	2019—Hospital	
Sg11	S	2020—Hospital	H5
	L15	2020—Hospital	
	L116	2021—Spa facility	SF
	L124	2011—Hotel	Ho2
	L125	2019—Hotel	Ho3
	L126	2020—Private Homes	P1

*The facilities are labeled as follow: H, hospital; HCF, health care facilities; C, company; SF, Spa facility; Ho, hotel; P, private homes*.

For each serogroup dataset (Sg1, Sg7, and Sg11), different clustering cut-off values were automatically calculated by the IR Biotyper® software. In particular, the clustering cut-off values used were 0.957 for the *Lp* Sg1, 0.687 for *Lp* Sg7, and 0.966 for *Lp* Sg11, showing a different homogeneity within the three different groups. Nevertheless, the clustering cut-off value was not taken into consideration for the exploratory analysis about relatedness of isolates since results of a reference method at the serogrouping level was not available; therefore the branch length was used to correlate the isolates.

Regarding *Lp* Sg1, the three isolates belonging to H1 as well as two out of three isolates of H3 complex showed very high relatedness. The third isolate of H3 complex showed no similarity with the others. The isolate belonging to the H2 complex was closely related to the H1 complex. The remaining isolates, belonging to other complexes, showed no relatedness. One isolate (L87) showed high similarity to the *Lp* Sg1 ATCC strain (Figure 4A).

Regarding *Lp* Sg7, since only two environmental isolates and one *Lp* ATCC were present in the dataset it was necessary to add a further isolate for technical reasons (a minimum number of isolates is necessary to perform LDA). The dendrogram shows clearly that the two environmental *Lp* Sg7 isolates are related, linked to the *Lp* Sg7 ATCC strain, and separated from the negative control (Figure 4B).

In the dendrogram elaborated for *Lp* Sg11, the isolates belonging to Ho2 and Ho3, as well as H5 isolate with *Lp* Sg11 ATCC shows showed a good degree of relatedness. The isolate from P1 displays a high relatedness with the SF isolate (Figure 4C).

## Discussion

The recent Italian outbreaks and the increment of *Lp* infections incidence in Italy and in other countries have demonstrated that a correct and fast characterization of isolates is important and necessary (Szewzyk et al., 2000; Faccini et al., 2020; Brunello et al., 2022). The diagnostic techniques used by clinical and environmental laboratories often lead to an absence or mis-classification of isolates, and as a consequence to the underestimation of the risk of infection, as documented by epidemiological data (Rota et al., 2021a). These issues require more relevant attention, also in the context of the pandemic event linked to Sars-Cov-2. Several epidemiological data (Rota et al., 2021b) other than recent literature (Dey and Ashbolt, 2021; Liang et al., 2021; Rhoads and Hammes, 2021) have already documented how the lockdown period and the absence of the preventive measures have led to an increase of *Lp* infection risk. Moreover, a mis-classification of cases was reported, which is attributable to the similarity of pneumonia symptoms with COVID-19 (Rota et al., 2021a). Therefore, the improvement or the introduction of techniques able to better identify the *Legionella* isolates could contribute to overcome the limit of traditional methods, increasing the discriminatory power and more accurately focusing on the public health choices.

The culture technique with a preliminary discrimination by the subculture in BCYE cys+ and cys- media is the first and essential step required for *Legionella* identification. In laboratory practice, *Lp* serogrouping is performed using the latex agglutination test, which consists of specific polyclonal antibodies available to identify the two major groups (Sg1 and Sg2–14), as well as individual serogroups. Although this technique gives rapid results and is relatively inexpensive, it nevertheless presents problems due to cross-reactions between the antibodies.

Recently, several studies demonstrated the advantages obtained by mass-spectroscopy techniques on bacteria classifications as well as the discriminatory power at species or strain levels (Li et al., 2019; Cordovana et al., 2021).

Among them, other than the widely recognized application of MALDI-TOF MS, the FTIR spectroscopy is being developed in routine species identification, but in the field of *Legionella* surveillance only few studies have been reported (Helm and Naumann, 1995).

Scientific literature have already shown how FTIR technique was able to discriminate and identify both Gram-positive and Gram-negative bacteria. The authors demonstrated its application on *Listeria monocytogenes, Streptococcus pneumoniae, Salmonella* spp, *Bifidobacterium* spp., and characterization both in clinical and food fields showing the potentiality of the technique as well as its limits (Helm et al., 1991b; Rebuffo-Scheer et al., 2007; Maity et al., 2013; Lasch et al., 2018; Deidda et al., 2021). Although Horbach et al. (1988) already demonstrated the application of FT-IR on *Legionella*, characterizing clinical strains involved in three cases of simultaneous infections, the application of FTIR on *Legionella* classification is poorly documented.

In this study, for the first time, the results of FT-IR technique using reference and environmental strains were compared to obtain reliable and standardized methods to quickly classify and correlate the isolates. Moreover, a new dataset with a large number of isolates was built to provide a new scheme of *Lp* serogroups classification that overcame the limit of software classifier provided by IR Biotyper®.

The results of this study show that FTIR technology can be used to correctly discriminate the Sg1, the most clinically relevant one, from the other *Lp* serogroups with high accuracy, showing advantages in terms of time and costs. The entire analysis requires about 2 hfor a 96-well plate, considering the time from beginning of sample preparation to results delivery. The interpretation of results is simple, quick, and non-operator dependent, and the storage of all data in the IR Biotyper® software allows the traceability of results that permits to reanalyze them in different time or match old results with new ones acquired over time. Moreover, the IR Biotyper® sensibility and specify reduce the laboratory costs compared to the traditional agglutination test.

Regarding the identification of other serogroups, the main isolates were classified inside the range 2–15 with moderate reliability (yellow score), and only four of them were classified as not reliable (red score). Despite the red score, the results obtained could be considered correct because our dataset was represented by isolates that had been already serogrouped with agglutination tests, and the single serogroup was already established. Also only one exception concerning two Sg7 isolates was observed, including the *Lp* ATCC strain, that was miss-classified as Sg1. Nevertheless, the Sg7 isolates were clearly separated from the other serogroups, when analyzed by PCA/LDA, and this apparent discrepancy could be explained with the assumption that the training set used to develop the classifier included no *Lp* Sg7 isolates, therefore the ANN could not possibly to learn to recognize the distinctive features of this serogroup, and consequently it was not able to recognize them when they were exhibited by unknown isolates.

Moreover, the cut-off value fixed by manufacturer classifier has been built with a limited dataset of isolates identified only between the main two groups: Sg1 vs. Sg2–15, without a specific serogrouping identification for the second one. This limit could explain the low score obtained during some identification, and the mis-classification returned for Sg7. Further and more focused investigation would be necessary to evaluate the potential of this technique for a discrimination of the other serogroups, especially for the more pathogenic ones like *Lp* Sg3 and Sg6. An extension of dataset with biological replicates grown on different culture conditions and coming from different geographical area could contribute to amplify the classifier with more features to set up a new cut-off, increasing the discrimination power of the instrument.

Similarity analysis by LDA delivered results coherent with results of the classifier, showing for the environmental strains a clear separation of Sg1, Sg7, and Sg11 from the other serogroups. By contrast, the ATCC strains appear well separated from each other, without any overlapping, while among the environmental isolates only 3 serogroups could be distinguished.

It is known that the ATCC strains are reference isolates that have been deposited in a collection and remain preserved. Therefore, compared to environmental strains, they do not undergo environmental stresses like the presence of disinfectants, microbial competition, and water temperature and flow that could affect their biochemical membrane components. Moreover, regarding the analysis performed, the different separations could be explained with the fact that each serogroup of the ATCC dataset is represented by one single isolate, tested as biological triplicate, while in the environmental dataset, each environmental serogroup is represented by more isolates. Therefore, the inter-serogroup variance is higher (as each isolate brings to the group its own variance) and the spectral area of the different serogroups may overlap, making them indistinguishable. Further investigations, using more isolates for each serogroup, a higher number of discriminative features (PCs and LDs), and a multi-step classification approach (e.g., a first differentiation in 1–7–11 vs. the rest, and a second step focused on “the rest”) could help in the separation of the other Sgs.

The HCA delivered promising preliminary results regarding correlation between the serogrouping already separated (e.g., Sg1, 7, and 11). The results clearly showed how isolates that come from several facilities and collected in different years were correlated with each other and regrouped in the same clade. These results should be confirmed and compared by molecular reference methods.

Our results support the proposal to use the IR Biotyper® system as an initial screening to distinguish the *Lp* serogroups isolates during clinical and environmental surveillance. Moreover, its association with MALDI-TOF MS, due to easy sample preparation and a rapid returned result, could improve the microbial identification and epidemiology investigation.

Currently the IR Biotyper® is able to distinguish only *Lp* Sg1 from all the other serogroups but, as demonstrated by our results, with greater accuracy. This is in accordance with the National and International Guidelines that recommended during the *Legionella* routine monitoring only the discrimination between *Lp* Sg1 and *Lp* Sg 2–15 (Italian National Institute of Health, 2015).

It is important to underline that the IR Biotyper® classifier is built with *Lp* Sgs isolates coming from Austria and Germany, while all our *Lp* Sgs have been isolated from facilities distributed on the Italian territory. Despite the different origin and year of isolation of the strains, it seems that the isolate's geographical origin did not affect the instrument's discriminatory power since our isolates were well identified and clustered.

This study opens up the possibility to use this technique to recognize and eventually correlate also isolates coming from different countries, underlying the role of its application also during epidemic events or travel-associated Legionnaires' disease cases. Moreover, in a first future step it will be essential to train the instrument (e.g., biological and technical replicates) with isolates that come from different geographical areas to overcome the limit of Sgs prevalence found in our study. When the classifier instrument will be trained with several Sgs', it could be possible to build a new classifier also for other *Legionella* species.

A second step will be the development of a new classifier starting from other isolates' features. Considering that the differentiation into 15 serogroups is given by different fatty acid components, it will be interesting to focus new studies on the evaluation of lipid profile adsorption. The IR Biotyper® is already able to perform it on other microorganisms, characterizing several differences on fatty acid and lipidic profile that are able to differentiate them also at species level (e.g., for Mycobacteria; Solntceva et al., 2021).

The limitation of the study could be attributed to the low numbers of isolates of some serogroups (e.g., *Lp* Sg7) with respect to the one most abundant (e.g., *Lp* Sg1), although this reflects the real prevalence of the isolates in the sampled area.

Moreover, an expertise is required to develop a new classifier, as we have tried to do in our study, to separate the isolates within the two main groups already defined by the classifier. This limit is exceeded when the user applied only the classifier present in the IR Biotyper® system that discriminates between *Lp* Sg1 and *Lp* 2–15.

In conclusion, the evaluation of applicability of the IR Biotyper® system is ongoing, and a prospective could be a multicentric study to test the method of reproducibility across different countries.

The findings of this study represent a starting point for the introduction of this technology for the rapid identification of *Lp* strains not only for diagnostic purposes and environmental surveillance, but mainly during outbreaks and clusters. By this promising and innovative tool both the implementation of control measures, risk assessment analysis, and the management of water distribution systems could be improved.

## Data Availability Statement

The original contributions presented in the study are included in the article/supplementary materials, further inquiries can be directed to the corresponding author/s.

## Author Contributions

SC and MRP conceived and designed the experiments, wrote the article, and performed data interpretation. MC performed data interpretation and provided all the information about the instrument classifier. MM, LG, and FM performed sample collection and the culture experiments. MRP, FB, and PDM performed the IR-Biotyper® analysis. MLR and MS supplied the communities of ATCC *Lp* strains other than to contribute to data interpretation and critical review of the manuscript. All the authors have read and agreed to the published version of the manuscript.

## Conflict of Interest

MC was employed by Bruker Daltonic GmbH (the manufacturer of IR-Biotyper®). The remaining authors declare that the research was conducted in the absence of any commercial or financial relationships that could be construed as a potential conflict of interest.

## Publisher's Note

All claims expressed in this article are solely those of the authors and do not necessarily represent those of their affiliated organizations, or those of the publisher, the editors and the reviewers. Any product that may be evaluated in this article, or claim that may be made by its manufacturer, is not guaranteed or endorsed by the publisher.
